# Isolated Liver Recurrence After Cytoreduction in High-Grade Serous Ovarian Carcinoma: Experience of a Tertiary Center in Turkey

**DOI:** 10.3390/jcm14061791

**Published:** 2025-03-07

**Authors:** Hande Esra Koca Yıldırım, İzzet Özgürlük, Burak Ersak, Dilek Yüksel, Eyüp Gökhan Turmuş, Baran Yeşil, Çiğdem Kılıç, Sevgi Koç, Nurettin Boran, Sadun Sucu, Caner Çakır

**Affiliations:** 1 Department of Gynecologic Oncology, Ümraniye Training and Research Hospital, 34764 İstanbul, Turkey; 2 Department of Obstetrics and Gynecology, Ankara Bilkent City Hospital, 06800 Ankara, Turkey; iozgurluk@gmail.com; 3 Department of Gynecologic Oncology, Ankara Etlik City Hospital, 06710 Ankara, Turkey; burakersak@gmail.com (B.E.); drdilekacar@hotmail.com (D.Y.); eyupturmus1903@gmail.com (E.G.T.); baranyesil@windowslive.com (B.Y.); caner4084@gmail.com (C.Ç.); 4 Department of Gynecologic Oncology, Etlik Zubeyde Hanim Women’s Health Training and Research Hospital, Faculty of Medicine, University of Health Sciences, 06100 Ankara, Turkey; cigdemkilic2002@gmail.com (Ç.K.); drskoc@hotmail.com (S.K.); nboranoglu@gmail.com (N.B.); 5 Department of Perinatology, Ankara Etlik City Hospital, 06710 Ankara, Turkey; medical.academic.sucu@gmail.com

**Keywords:** high-grade serous ovarian cancer, hepatic metastases

## Abstract

**Background:** Serous epithelial ovarian cancer is typically diagnosed at an advanced stage and often recurs following treatment. Isolated organ recurrence is rare in this disease, making treatment planning a critical decision. Therefore, we investigated the survival rates of patients who developed isolated liver recurrence. **Methods**: The entire cohort included patients who underwent cytoreductive surgery between January 1993 and December 2020. We evaluated patients who completed primary chemotherapy after cytoreductive surgery based on their status of isolated liver recurrence. We created two groups: patients with isolated parenchymal recurrence and patients with isolated capsular recurrence. Staging was based on the International Federation of Gynecology and Obstetrics (FIGO) 2014 staging criteria. For patients treated before 2014, cancer staging was adapted to the FIGO 2014 system based on a surgical and pathological assessment. **Results:** The mean ages of patients with liver capsule and parenchymal recurrence at the time of primary surgery were 47 ± 10.6 and 49 ± 8.9 years, respectively. The median recurrence of patients with capsular recurrence was 13 (2–70) months. In patients with parenchymal recurrence, the duration was 10 months (4–80) and was statistically insignificant. While survival was 41.5 (5–120) months in patients with capsular recurrence, it was 34 (12–120) months in patients with parenchymal recurrence, but there was no statistical difference. **Conclusions:** In our 27 years’ of experience with EOC management, we have studied patients with isolated liver recurrences. The finding that either capsular or parenchymal liver recurrence has no significant impact on overall survival suggests that both types of recurrence can be managed with similar treatment and follow-up approaches. This observation could simplify patient management and improve outcomes by allowing clinicians to focus on optimal surgical and systemic treatment strategies rather than the anatomic pattern of recurrence.

## 1. Introduction

Ovarian cancer is the most lethal cancer of the female genital system, and the most common histopathologic type is epithelial high-grade serous carcinoma. In this disease, for which there is no effective screening method, patients are diagnosed at an advanced stage (IIIC-IVB) [1]. Primary treatment consists of maximal cytoreduction followed by platinum-based chemotherapy and is one of the most important factors for survival [2]. Based on the literature, an evaluation by a specialized gynecologic oncologist is recommended to determine the best surgical approach and to perform the primary surgical approach [3]. Preoperative imaging is very valuable in the decision-making phase and in planning the operation. Preoperative imaging makes it possible to estimate the duration of the operation and to determine the need for intraoperative consultant specialists in advance. Today, computed tomography (CT) is the standard for the preoperative assessment of patients with ovarian cancer. However, the success of advanced magnetic resonance imaging (MRI) cannot be ignored, particularly in the assessment of small peritoneal lesions in difficult-to-resect areas. Positron emission tomography (PET)-CT imaging in the preoperative setting is currently limited, while the use of the new hybrid PET-MRI technique is still under investigation [4]. Various scoring systems are used in the treatment of patients to decide whether to undergo primary cytoreductive surgery or neoadjuvant chemotherapy. Surgical resectability can be assessed according to the Leuven criteria, the Essen criteria or the Fagotti scoring system [5]. The Essen criteria are based on intraoperative findings to determine the maximum number of patients who can benefit from primary cytoreductive surgery. Surgery begins with the detection of biopsy-proven stage 3c and 4 epithelial ovarian, fallopian tube or peritoneal carcinoma [6].

Studies have shown that complete resection is the only independent factor that significantly improves overall survival and disease-free survival, regardless of the surgical strategy [7]. Despite planned and effective primary cytoreductive surgery and subsequent platinum-based chemotherapy, recurrence rates are quite high [8].

However, most relapses occur within the first 5 years, and although these relapses often occur intra-abdominally, they are rarely organ-specific [3]. In relapse, secondary and even tertiary cytoreduction is performed in suitable patients, with chemotherapy and palliation in unsuitable patients [9].

Recurrent ovarian carcinoma is rarely specific to a single organ and may recur in the liver. However, it is commonly seen in the liver along with widespread intra-abdominal recurrence in patients who have died from the disease [10]. In serous ovarian cancer, liver recurrence can affect survival rates differently depending on the type of metastasis. While hepatic parenchymal invasion does not appear to have a negative impact on survival, hematogenous metastases are associated with a shorter survival rate [11]. Secondary cytoreduction has become increasingly important in patients with isolated liver recurrence, but it has been difficult to reach a consensus on the optimal treatment [12]. Complete cytoreductive surgery, including liver resection, has been shown to be a viable approach with or without additional HIPEC (Hyperthermic Intraperitoneal Chemotherapy) and may offer a survival benefit for patients with advanced and/or recurrent ovarian cancer [13]. In selected patients, liver resection during cytoreduction can improve the survival rate in patients with advanced and recurrent ovarian cancer [14].

Our aim in this study was to investigate the survival rate of patients who develop isolated liver recurrence in patients who have undergone primary cytoreduction and platinum-based chemotherapy for serous ovarian cancer.

## 2. Materials and Methods

The study was initiated with the approval of the hospital’s ethics committee and was conducted in accordance with the Declaration of Helsinki (approval number: AESH-BADEK-EZH-BADEK-06, date: 3 July 2024). The patients’ data were obtained by searching the electronic medical record.

This retrospective cohort study included 1247 patients who underwent cytoreductive surgery with a diagnosis of ovarian cancer between January 1993 and December 2020. All patients underwent total abdominal hysterectomy, bilateral salpingo-oophorectomy, total omentectomy, systematic lymphadenectomy and cytoreductive surgery. Patients with a pathological finding of serous ovarian carcinoma and isolated liver recurrence after primary cytoreductive surgery were examined. Patients who underwent liver resection as part of primary cytoreductive surgery or due to persistent disease were excluded.

Synchronous tumors and tumors with non-epithelial and serous components were excluded from the study group. In addition, patients with extrahepatic tumors who underwent imaging and secondary cytoreduction were excluded from the study. Patients with other malignant tumors, patients who had received neoadjuvant chemotherapy, patients who had undergone surgery at other institutions and patients who had not undergone optimal cytoreduction were also excluded from the study. The evaluation of primary cytoreduction was based on the Essen criteria [6]. All surgical procedures were performed by experienced gynecologic oncologic surgeons, and all patients received 6 cycles of taxane-platinum-based chemotherapy. The study cohort consisted of 1247 patients at the beginning of the study; 145 patients were excluded from the study because their medical records were incomplete. Of the 1102 patients, 881 (80%) underwent R0 cytoreductive surgery. Recurrence occurred in 466 (53%) of the 881 patients who underwent R0 cytoreductive surgery. Of the 466 patients who had a recurrence after primary cytoreductive R0 surgery, 45 (9.6%) had isolated liver recurrence. Two groups were included in the study: 18 patients with isolated parenchymal recurrence and 27 patients with isolated capsular recurrence.

Patients who showed a complete clinical response after initial treatment were followed up at 3-month intervals for the first 2 years, 6-month intervals for up to 5 years and 1-year intervals after that, during which a pelvic examination, abdominal pelvic ultrasound, complete blood count, blood chemistry and serum tumor markers were performed. A chest X-ray was performed annually. In suspected cases, thoracic and abdominal computed tomography (CT) and magnetic resonance imaging (MRI) were performed.

Staging was based on the International Federation of Gynecology and Obstetrics (FIGO) 2014 staging criteria. For patients treated prior to 2014, cancer staging was adapted to the FIGO 2014 system based on a surgical and pathological assessment. Disease progression during first-line adjuvant chemotherapy was defined as “refractory disease”. If a partial clinical response or stable disease was observed, the same adjuvant chemotherapy regimen was continued. During this adjuvant chemotherapy, patients were re-evaluated and classified as having a complete clinical response or refractory disease. Radiologic (detection of new lesions with modern imaging techniques) and laboratory (increase in CA-125 levels) recurrence of the disease in patients with a complete clinical response was classified as “recurrent disease”. Overall survival (OS) was accepted as the time from initial surgery to death due to the disease or the last follow-up visit. Disease-free survival (DFS) was defined as the period from initial surgery to proven recurrence or refractory disease with a clinical examination and/or radiologic imaging or the period from initial surgery to the last follow-up visit with no refractory disease/recurrence did not occur. Post-recurrence survival (PRS) was defined as the time from disease recurrence to death.

### Statistical Analysis

SPSS 20.0 (SPSS Inc., Chicago, IL, USA) was used for data management and statistical analysis. Comparisons between groups were performed using the ×2 test and the Mann–Whitney U test. Descriptive statistics were expressed as the mean ± standard deviation or median (min–max) for continuous variables and number/percentage for categorical variables. Survival outcomes were calculated using the Kaplan–Meier method. Survival curves were compared using the log-rank test. *p*-values less than 0.05 were considered significant.

## 3. Results

In our study, recurrence occurred in 466 (53%) of the 881 patients who underwent R0 cytoreductive surgery. Our cohort of 45 patients was examined in two groups: 18 patients with isolated parenchymal recurrence and 27 patients with isolated capsular recurrence. The mean age of patients with liver capsule and parenchymal recurrence at the time of primary surgery was 47 ± 10.6 and 49 ± 8.9 years, respectively. The mean number of lymph nodes removed was 59 ± 20 and 79 ± 44. The median numbers of affected lymph nodes were 5 (1–39) and 9 (1–57). While 7 (25.9%) of the patients with capsular recurrence had ascites, no ascites was observed in 20 patients. In the patients with parenchymal recurrence, ascites was present in 8 patients (44.4%) and absent in 10 patients (55.6%). Peritoneal cytology was positive in 17 (63%) of the patients with capsular recurrence, while it was negative in 5 (18.5%), and no peritoneal cytology samples were obtained in 5 patients. In patients with parenchymal recurrence, peritoneal cytology was positive in 10 patients (55.6%), negative in 4 patients (22.2%) and no sample was taken in 4 patients. Total omentectomy was performed in all patients, and the status of omental involvement was determined. In the patients with capsular recurrence, this was observed in 21 patients (77.8%) and was absent in 6 patients (22.2%). In the patients with parenchymal recurrence, 12 (66.7%) patients had omental involvement, while 6 patients (33.3) did not, and no statistical difference was observed between the groups.

Splenectomy was performed in 8 (29.6%) of the patients with capsular recurrence and 12 (66.7%) of the patients with parenchymal recurrence due to tumor involution, and it was statistically found that more tumors were present in the spleen in the patients with parenchymal recurrence (n = 12, 66.7%, *p* = 0.032). In addition, diaphragmatic stripping was performed in 9 (33.3%) of the patients with capsular recurrence due to diaphragmatic involvement, while this was the case in only 1 patient (5.6%) of the patients with parenchymal recurrence, and a statistical difference was found. Bowel resections due to the tumor were not statistically significant in either group (Table 1).

Considering the staging of the patients at primary surgery, among the patients with capsular recurrence, 1 patient was in stage 1C, 2 patients in stage 2B, 3 patients in stage 3B, 19 patients in stage 3C, 1 patient in stage 4A and 1 patient in stage 4B. Of the patients with parenchymal recurrence, 1 patient was stage 1B, 2 patients were stage 2A, 4 patients were stage 3B, 10 patients were stage 3C and only 1 patient was stage 4B. There was no statistical difference between the two groups (Table 2).

**Figure 1:**
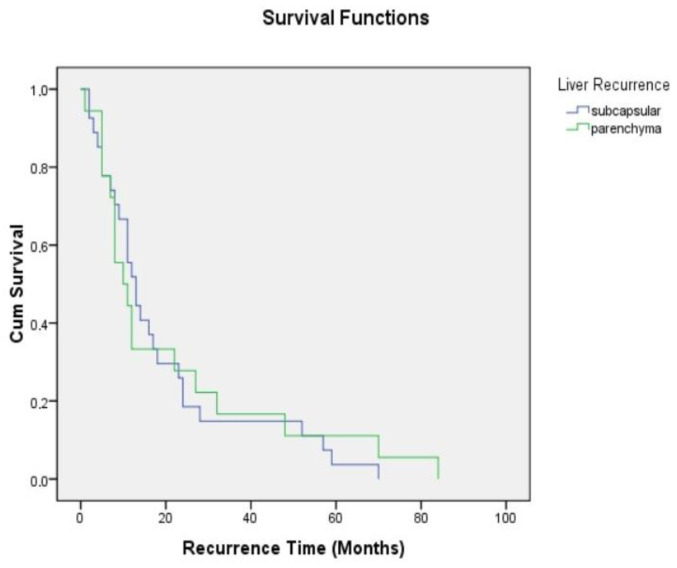
DFS: Disease-free survival.

**Figure 2:**
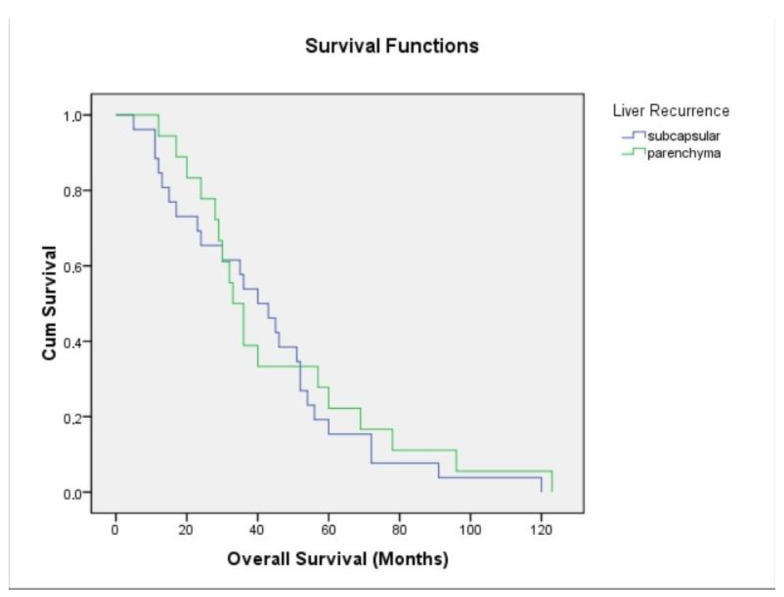
OS: overall survival.

The median recurrence of patients with capsular recurrence was 13 (2–70) months. In patients with parenchymal recurrence, the duration was 10 months (4–80) and was statistically insignificant (Figure 1). The serum CA-125 level was higher in patients with parenchymal recurrence (median = 454 IU/mL (10–2724), *p* = 0.457). For survival, while survival was 41.5 (5–120) months in patients with capsular recurrence, it was 34 (12–120) months in patients with parenchymal recurrence, but there was no statistical difference (Figure 2). In the treatment of recurrence, secondary cytoreduction was performed in all but one patient with capsular recurrence, while chemotherapy was performed in one patient. In the patients with parenchymal recurrence, only chemotherapy was performed in 16 patients because they had multiple recurrences, while liver resection was performed in 2 patients (Table 3).

## 4. Discussion

This study investigated the survival of patients with isolated liver recurrence in patients who had reached the maximum surgery rate for primary cytoreductive surgery for serous ovarian cancer and received platinum-based chemotherapy. When the survival rates of patients with parenchymal and capsular metastases were evaluated in this study, it was found that there was no difference in survival between patients with parenchymal metastases and capsular metastases when liver resection could not be performed in patients with multiple liver metastases. This is the first article to investigate isolated liver metastases after primary cytoreductive surgery and platinum-based chemotherapy for high-grade serous ovarian cancer.

Miklos Acs et al. have shown that liver resection may provide a survival benefit in patients with primary and recurrent ovarian cancer who have undergone liver resection as part of primary cytoreduction [13]. Roh, H.J. et al. published their experience using hepatic resection as part of secondary cytoreductive surgery for metachronous liver metastases from ovarian cancer. Prognostic factors associated with improved survival in the data from 18 patients included less abdominal than pelvic disease (38 vs. 11 months, *p* = 0.032), optimal cytoreduction (40 vs. 9 months, *p* = 0.0004) and a negative margin status of hepatic resection (40 vs. 9 months, *p* = 0.0196). The median overall survival after liver resection was 38 months (range 3–78 months) [15]. In studies of hepatic resection for recurrent metachronous ovarian cancer, Merideth MA et al. found that in ovarian cancer, the survival rate of patients who underwent liver resection was similar to that of patients without liver disease but with a similar volume of residual disease [16].

The time elapsed after the completion of primary platinum-based chemotherapy and the form of recurrent disease are considered important factors in the prognosis of women with recurrent ovarian cancer. It has been demonstrated in the literature that there is a direct correlation between the duration of the platinum-free interval (PFI) and survival after relapse [17]. In our study cohort, 881 out of 1102 patients underwent R0 cytoreductive surgery. Recurrence was observed in 466 out of 881 patients. Our recurrence rate of 53% aligns with previous reports in the literature, which range between 45 and 60% [18]. The median time of disease recurrence in 421 patients was 21 months, while the median survival time was 52 months. In our study, patients with capsular recurrence were treated with secondary cytoreduction, while 16 patients with parenchymal metastases had multiple recurrences, so chemotherapy was chosen in the patients with parenchymal metastases, and liver resection was performed in only 2 patients. Our study found that liver recurrences occurred within 21.26 months of the initial treatment. Consistent with previous research, capsular recurrences lasted an average of 13 months (2–70 months) and parenchymal recurrences lasted an average of 10 months (4–80 months). One patient had a recurrence 6 months after the end of primary chemotherapy. The liver lesion prompted the decision to operate. During the operation, the patient underwent a left hepatic lobectomy. The patient died 11 months after this operation. The other patient had a recurrence 23 months after the end of primary chemotherapy. The patient who underwent a right liver segmentectomy died 8 months after the operation.

Epithelial ovarian carcinomas spread mainly through trans-coelomic metastases implanted in the peritoneal cavity [19]. In our study, it was observed that patients with tumors in the diaphragm during primary cytoreduction surgery had more liver recurrences in the capsule during secondary surgery.

In our study, splenectomy was performed in 20 patients as part of primary cytoreduction. In these patients, isolated liver recurrences in the parenchyma were observed to be more frequent (n = 12, 66.7%, *p* = 0.032). The spread of the tumor originating from the venous system may have played a role in this pathogenesis process. We know that the disease is more widespread and that 3C, in particular, is a heterogeneous group.

In patients who underwent rectosigmoid resection as part of primary cytoreduction surgery, although not statistically significant, more frequent metastasis of the liver parenchyma was observed (n = 6, 33.3%, *p* = 0.166).

This study has several limitations. First, it was retrospective. The number of patients with isolated liver metastases after treatment was small, so subgroups were limited. The evolution of therapeutic approaches during the study period led to some heterogeneity in treatment strategies. Our cohort included patients who received taxane-platinum-based chemotherapy after cytoreductive surgery. However, the statistics for the refractory and resistant groups were not included. The strength of this study is that the patient data were analyzed over a long period of 27 years. It is the first study to investigate isolated liver metastases after primary cytoreduction surgery and platinum-based chemotherapy for high-grade serous ovarian cancer.

## 5. Conclusions

In the present study, patients with serous ovarian carcinoma who had undergone primary cytoreductive surgery and received platinum-based chemotherapy were evaluated for isolated liver recurrences observed during follow-up. In patients who had undergone splenectomy during primary cytoreduction, liver recurrence was predominantly found to be parenchymal metastases, whereas patients who had undergone diaphragmatic stripping were more likely to have capsular liver recurrence. The similar impact of capsular or parenchymal isolated liver recurrence on overall survival suggests that both recurrences can be managed with similar treatment and follow-up approaches. This observation could simplify patient management and improve outcomes by allowing clinicians to focus on optimal surgical and systemic treatment strategies rather than anatomic recurrence patterns. However, further studies with larger numbers of patients are needed to confirm the results and generalize to a larger sample.

## Figures and Tables

**Table 1:** Surgical and pathological characteristics of patients.

Variables	Capsule (n:27)	Parenchyma (n:18)	*p*
Age at surgery, mean ± SD, years	47 ± 10.6	49 ± 8.9	0.603
Number of LNs removed, mean ± SD	59 ± 20	79 ± 44	0.148
Number of lymph nodes involved, median (min–max)	5 (1–39)	9 (1–57)	0.599
CA 125 (IU/mL)	416 (7–10,300)	454 (10–2724)	0.457
Ascites [n (%)]			0.333
Preent	7 (25.9)	8 (44.4)	
Absent	20 (74.1)	10 (55.6)	
Peritoneal cytology [n (%)]			0.712
Positive	17 (63)	10 (55.6)	
Negative	5 (18.5)	4 (22.2)	
Not received	5 (18.5)	4 (22.2)	
Omental involvement [n (%)]			0.499
Positive	21 (77.8)	12 (66.7)	
Negative	6 (22.2)	6 (33.3)	
Splenectomy [n (%)]			0.032
Performed	8 (29.6)	12 (66.7)	
Not performed	19 (70.4)	6 (33.3)	
Diaphragm striping [n (%)]			0.034
Performed	9 (33.3)	1 (5.6)	
Not performed	18 (67.7)	17 (94.6)	
Small bowel resection [n (%)]			>0.05
Performed	1 (3.7)	-	
Not performed	26 (96.3)	18 (100)	
Large bowel resection [n (%)]			>0.05
Performed	1 (3.7)	-	
Not performed	26 (96.3)	18 (100)	
Rectosigmoid resection [n (%)]			0.166
Performed	4 (14.8)	6 (33.3)	
Not performed	23 (85.2)	12 (66.7)	
Appendectomy [n (%)]			>0.05
Performed	21 (77.8)	14 (77.8)	
Not performed	5 (22.2)	4 (22.2)	

Abbreviations: SD, standard deviation, LN, lymph node. Data are presented as the mean ± SD or median (interquartile range), as appropriate. A *p*-value of <0.05 was considered statistically significant.

**Table 2:** FIGO 2014 stage.

Stage	Capsule (n:27)	Parencyma (n:18)	*p*
1A	-	-	0.265
1B		1	
1C	1	-	
2A	-	2	
2B	2	-	
3A	-	-	
3B	3	4	
3C	19	10	
4A	1	-	
4B	1	1	

Abbreviations: FIGO, International Federation of Gynecology and Obstetrics.

**Table 3:** Survival and recurrence treatment.

Variables	Capsule (n:27)	Parencyma (n:18)	*p*
Median time to liver recurrence (months)	13 (2–70)	10 (4–80)	0.745
Serum Ca–125 level at liver recurrence, median (range), U/mL	73 (11–2350)	124 (24–3290)	0.018
Recurrence treatment			<0.001
Only CT	1	16 *	
Surgery +CT	26	2 **	
Follow-up median (min–max)	41.5 (5–120)	34 (12–120)	0.730

Abbreviations: CT, chemotherapy * Multiple, ** Soliter tumor (Segmentectomy)**.** Data are presented as the mean ± SD or median (interquartile range), as appropriate. A *p*-value of <0.05 was considered statistically significant.

## Data Availability

The data presented in this study are available on request from the corresponding author. The data are not publicly available due to [privacy/ethical] restrictions.

## References

[1] Siegel R., Naishadham D., Jemal A. (2013). Cancer statistics, 2013. CA Cancer J. Clin..

[2] Griffiths C.T. (1975). Surgical resection of tumor bulk in the primary treatment of ovarian carcinoma. Natl. Cancer Inst. Monogr..

[3] Armstrong D.K., Alvarez R.D., Backes F.J., Bakkum-Gamez J.N., Barroilhet L., Behbakht K., Berchuck A., Chen L.M., Chitiyo V.C., Cristea M. (2022). NCCN Guidelines^®^ Insights: Ovarian Cancer, Version 3.2022. J. Natl. Compr. Cancer Netw..

[4] Rizzo S., Del Grande M., Manganaro L., Papadia A., Del Grande F. (2020). Imaging before cytoreductive surgery in advanced ovarian cancer patients. Int. J. Gynecol. Cancer Off. J. Int. Gynecol. Cancer Soc..

[5] Vergote I., du Bois A., Amant F., Heitz F., Leunen K., Harter P. (2013). Neoadjuvant chemotherapy in advanced ovarian cancer: On what do we agree and disagree?. Gynecol. Oncol..

[6] Lheureux S., Gourley C., Vergote I., Oza A.M. (2019). Epithelial ovarian cancer. Lancet.

[7] Delga B., Classe J., Houvenaeghel G., Blache G., Sabiani L., Hajj E., Andrieux N., Lambaudie E. (2020). 30 Years of Experience in the Management of Stage III and IV Epithelial Ovarian Cancer: Impact of Surgical Strategies on Survival. Cancers.

[8] Akgöl S., Aktürk E., Özaydın İ., Ölmez F., Karakaş S., Oğlak S., Budak A., Ölmez Ö., Budak M., Akbayır Ö. (2021). Serous Epithelial Ovarian Cancer: Retrospective Analysis of 260 Cases. Aegean J. Obstet. Gynecol..

[9] Harter P., Sehouli J., Vergote I., Ferron G., Reuss A., Meier W., Greggi S., Mosgaard B.J., Selle F., Guyon F. (2021). Randomized Trial of Cytoreductive Surgery for Relapsed Ovarian Cancer. N. Engl. J. Med..

[10] Rose P.G., Piver M.S., Tsukada Y., Lau T.S. (1989). Metastatic patterns in histologic variants of ovarian cancer. An autopsy study. Cancer.

[11] O’Neill A., Somarouthu B., Tirumani S., Braschi-Amirfarzan M., Van Den Abbeele A., Ramaiya N., Shinagare A. (2017). Patterns and Prognostic Importance of Hepatic Involvement in Patients with Serous Ovarian Cancer: A Single-Institution Experience with 244 Patients. Radiology.

[12] Adam R., Chiche L., Aloia T., Elias D., Salmon R., Rivoire M., Jaeck D., Saric J., Le Treut Y.P., Belghiti J. (2006). Hepatic resection for noncolorectal nonendocrine liver metastases: Analysis of 1452 patients and development of a prognostic model. Ann. Surg..

[13] Acs M., Herold Z., Neumann L., Slowik P., Evert K., Gurok S., Panczel I., Barna A., Dank M., Szász A. (2024). Surgical Treatment and Outcome of Ovarian Cancer Patients with Liver Metastases: Experience of a Tertiary Hepatic and Peritoneal Surface Malignancy Center. Anticancer Res..

[14] Gasparri M., Grandi G., Bolla D., Gloor B., Imboden S., Panici P., Mueller M., Papadia A. (2016). Hepatic resection during cytoreductive surgery for primary or recurrent epithelial ovarian cancer. J. Cancer Res. Clin. Oncol..

[15] Roh H.J., Kim D.Y., Joo W.D., Yoo H.J., Kim J.H., Kim Y.M., Kim Y.T., Nam J.H. (2011). Hepatic resection as part of secondary cytoreductive surgery for recurrent ovarian cancer involving the liver. Arch. Gynecol. Obstet..

[16] Merideth M.A., Cliby W.A., Keeney G.L., Lesnick T.G., Nagorney D.M., Podratz K.C. (2003). Hepatic resection for metachronous metastases from ovarian carcinoma. Gynecol. Oncol..

[17] Markman M., Markman J., Webster K., Zanotti K., Kulp B., Peterson G., Belinson J. (2004). Duration of response to second-line, platinum-based chemotherapy for ovarian cancer: Implications for patient management and clinical trial design. J. Clin. Oncol..

[18] Wang Q., Zheng Y., Wang P., Zhang J., Liu H., Li Q., Yin R., Bian C., Peng H., Peng Z. (2021). The prognostic factor for recurrence in advanced-stage high-grade serous ovarian cancer after complete clinical remission: A nested case-control study. J. Ovarian Res..

[19] Tan D.S., Argarwal R., Kaye S.B. (2006). Mechanisms of transcoelomic metastasis in ovarian cancer. Lancet Oncol..

